# Exposure to Phthalates and Breast Cancer Risk in Northern Mexico

**DOI:** 10.1289/ehp.0901091

**Published:** 2009-12-09

**Authors:** Lizbeth López-Carrillo, Raúl U. Hernández-Ramírez, Antonia M. Calafat, Luisa Torres-Sánchez, Marcia Galván-Portillo, Larry L. Needham, Rubén Ruiz-Ramos, Mariano E. Cebrián

**Affiliations:** 1 National Institute of Public Health, Cuernavaca, Morelos, México; 2 Division of Laboratory Sciences, National Center for Environmental Health, Centers for Disease Control and Prevention, Atlanta, Georgia, USA; 3 Departamento de Toxicología, Centro de Investigación y de Estudios Avanzados del Instituto Politécnico Nacional, Mexico City, México

**Keywords:** breast cancer, case–control study, endocrine disruptors, environment, Mexico, phthalates, risk assessment, urinary metabolites

## Abstract

**Background:**

Phthalates, ubiquitous environmental pollutants that may disturb the endocrine system, are used primarily as plasticizers of polyvinyl chloride and as additives in consumer and personal care products.

**Objectives:**

In this study, we examined the association between urinary concentrations of nine phthalate metabolites and breast cancer (BC) in Mexican women.

**Methods:**

We age-matched 233 BC cases to 221 women residing in northern Mexico. Sociodemographic and reproductive characteristics were obtained by direct interviews. Phthalates were determined in urine samples (collected pretreatment from the cases) by isotope dilution/high-performance liquid chromatography coupled to tandem mass spectrometry.

**Results:**

Phthalate metabolites were detected in at least 82% of women. The geometric mean concentrations of monoethyl phthalate (MEP) were higher in cases than in controls (169.58 vs. 106.78 μg/g creatinine). Controls showed significantly higher concentrations of mono-*n*-butyl phthalate, mono(2-ethyl-5-oxohexyl) phthalate, and mono(3-carboxypropyl) phthalate (MCPP) than did the cases. After adjusting for risk factors and other phthalates, MEP urinary concentrations were positively associated with BC [odds ratio (OR), highest vs. lowest tertile = 2.20; 95% confidence interval (CI), 1.33–3.63; *p* for trend < 0.01]. This association became stronger when estimated for premenopausal women (OR, highest vs. lowest tertile = 4.13; 95% CI, 1.60–10.70; *p* for trend < 0.01). In contrast, we observed significant negative associations for monobenzyl phthalate (MBzP) and MCPP.

**Conclusions:**

We show for the first time that exposure to diethyl phthalate, the parent compound of MEP, may be associated with increased risk of BC, whereas exposure to the parent phthalates of MBzP and MCPP might be negatively associated. These findings require confirmation.

Phthalates are endocrine disruptors that have shown effects on reproductive health and development. Infertility, decreased sperm counts, cryptorchidism, reproductive tract malformations, hypospadias, and testicular tumors, as well as reduction on testosterone levels, anogenital distance, and reproductive organ weights and nipple retention, have been described both in animal and human studies (National Research Council 2008). Exposure to various phthalates in adult men has been associated with altered semen quality, reduced concentration of certain sexual and thyroid hormones, reduced pulmonary function, and increases in certain metabolic syndrome markers (Hauser and Calafat 2005; Swan 2008). In boys, prenatal exposure to some phthalates has been associated with a reduced anogenital distance (Swan et al. 2005). Exposure to phthalates before birth has been related to gestational age, and exposure during childhood has been associated with respiratory problems, asthma, and allergies (Adibi et al. 2009; Hauser and Calafat 2005; Swan 2008; Wolff et al. 2008).

Several phthalates, such as diethyl phthalate (DEP) and dibutyl phthalate (DBP), are widely used, especially in cosmetic and personal care products for infants, children, and adults. Other phthalates, including diisobutyl phthalate (DiBP), butylbenzyl phthalate (BBzP), di(2-ethylhexyl) phthalate (DEHP), and di-*n*-octyl phthalate (DOP), can be used as plasticizers in the manufacture of flexible vinyl plastic in consumer products, flooring and wall coverings, food contact applications, and medical devices. Phthalates can also be used as solvents in combination with other plasticizers in floor coverings as well as in some cosmetics and pharmaceutical products (David and Gans 2003; Frederiksen et al. 2007; Meeker et al. 2007).

Some phthalates can be absorbed through the skin (Janjua et al. 2007); in addition, they can be ingested, because they can migrate from wrappers and containers to foods (Wormuth et al. 2006). The metabolism of most phthalates in humans occurs first by hydrolysis of one ester bond to form the hydrolytic phthalate monoesters. Some phthalates may undergo a phase I biotransformation in which oxidative metabolites are formed. Both monoester and oxidative metabolites may react with glucuronic acid in a phase II biotransformation to form their respective glucuronide conjugates. The phase II conjugation facilitates urinary excretion of the phthalate metabolites (Silva et al. 2003). The urinary concentrations of phthalate metabolites have been used extensively to assess exposure to phthalates in epidemiologic studies. The metabolism of phthalates was recently reviewed by Frederiksen et al. (2007) and the National Research Council (2008).

Women may be at a higher risk than men for potential adverse health effects of phthalates due to phthalate exposure through cosmetics use. However, potential effects of phthalate exposure have been documented primarily in males. Health effects in women have been scarcely characterized, and studies have been limited to endometriosis (Cobellis et al. 2003; Reddy et al. 2006a, 2006b) and thyroid hormone changes (Huang et al. 2007).

Breast cancer (BC) is hormone dependent. Less than 25% of patients have a history of early menarche, later age at first childbirth, nulliparity, family history of BC, or history of benign breast biopsy (Rockhill et al. 1998); however, in most cases the causes of breast tumors are unknown, and environmental and genetic factors may play a role. The purpose of this study was to examine the association between exposure to six phthalates, estimated from urinary concentrations of nine selected phthalate metabolites, and the risk of BC among a group of northern Mexican women.

## Materials and Methods

### Study population

From March 2007 to August 2008 an epidemiologic population-based case–control study was performed in the northern states of Mexico (Baja California Norte, Chihuahua, Coahuila, Durango, Nuevo León, Sonora, and Tamaulipas). Cases were identified from 25 tertiary hospital units, which covered 90% of the study area population, including Health Department (Secretaría de Salud), Social Security (Instituto de Seguridad y Servicios Sociales), and State Workers’ Social Security (Instituto de Seguridad y Servicios Sociales de los Trabajadores del Estado) hospitals, as well as university health centers. We identified 233 patients with histopathologically confirmed BC, with a minimal age of 18, without any other cancer history, and with a residency period of > 1 year in the study area (inclusion criteria).

The controls consisted of 221 healthy women that were matched 1:1 by age (± 5 years) and residency with the index case. Controls were identified through the master sample framework used in the Health Department (Secretaría de Salud) national surveys (Tapia-Conyer et al. 1992). A housing list representative of the study area was obtained, and it included an access sketch to facilitate the location of the probabilistically selected homes. In the cases where there was more than one eligible woman in a home, one participant was randomly chosen. Conversely, if no eligible woman was found in a household, or if she declined participation in the study, a new home was systematically located according to the survey procedures employed in other studies. This study was approved by the Mexico National Institute of Public Health ethical committee. The involvement of the Centers for Disease Control and Prevention (CDC) laboratory was limited and determined not to constitute engagement in human subjects research.

### Interviews and urine samples

With their informed consent, women were directly interviewed by personnel trained to carry out structured interviews. Information was obtained about sociodemographic characteristics; clinical, reproductive (e.g., parity, lactation), and family medical history; dietary patterns; and anthropometric measures (e.g., weight, height). Patients were interviewed after their diagnosis, before any kind of treatment (average time from diagnosis ~ 2 months). Response rates (participants/eligible) were 94.8% for cases and 99.5% for controls.

A first morning void urine sample of each woman was collected in a sterile disposable polypropylene urine collection cup (polypropylene plastics have not been reported to contain detectable levels of phthalates). Among all cases, urine samples were obtained before any kind of treatment (including surgery and radiation therapy) was performed to exclude the possibility that phthalate exposure may have been influenced by cancer treatment. An aliquot of 4 mL of urine was prepared in a Cryovial (Simport Scientific, Beloeil, QC, Canada) and stored frozen at or below −20°C until shipment to the CDC. At the CDC, samples were kept frozen at −40°C until they were analyzed.

### Assessment of phthalate metabolite urinary concentrations

The urinary concentrations of nine phthalate metabolites [monoethyl phthalate (MEP), mono-*n*-butyl phthalate (MBP), monoisobutyl phthalate (MiBP), monobenzyl phthalate (MBzP), mono(3-carboxypropyl) phthalate (MCPP), and four metabolites of DEHP: mono(2-ethylhexyl) phthalate (MEHP), mono(2-ethyl-5-hydroxyhexyl) phthalate (MEHHP), mono(2-ethyl-5-oxohexyl) phthalate (MEOHP), and mono(2-ethyl-5-carboxypentyl) phthalate (MECPP)] were measured in the analytical organic toxicology laboratory of the CDC according to methodology published elsewhere that includes solid-phase extraction coupled with high-performance liquid chromatography/isotope dilution/tandem mass spectrometry (Kato et al. 2005).

### Statistical analysis

First-hand confirmation of the association of known BC risk factors was obtained through nonconditional logistic regression models. A linear trend between BC and parity, age at first birth, lactation, and body mass index was assessed by incorporating each factor as a continuous variable. Correlation coefficients were calculated between phthalates and BC risk factors among controls. Alpha level was set at 95%, and statistical significance was *p* < 0.05.

Phthalate metabolite urinary concentrations were adjusted for urine dilution by creatinine levels according to a previously detailed methodology (Barr et al. 2005) and transformed to a logarithmic scale. Phthalate metabolite concentrations below the limit of detection (LOD) were assigned a value equal to half the LOD (LOD/2) for the analysis. Correlations between metabolite concentrations were estimated. The geometric means of these concentrations were compared between cases and controls with the Student *t*-test and stratified by menopausal status.

Tertiles of phthalate exposure were created according to the observed distribution of urinary concentrations of the metabolites in the controls. To evaluate their potential association to BC, two multivariate logistic models were run for each phthalate metabolite. One model was adjusted for age, and the variables that were significantly correlated with any of the phthalate metabolite concentrations among controls: age of menarche, parity, and menopause. The second model for MEP, MBP, MiBP, MBzP, and MCPP was adjusted for the previous variables and the sum of DEHP metabolites; similarly, the second model for DEHP was adjusted for MEP, MBP, MiBP, MBzP, and MCPP. The models that reached the statistical level of significance were further stratified by menopausal status. Median values were assigned to phthalate metabolite concentrations tertiles, and they were modeled as continuous variables to estimate trend *p*-values. Active smoking did not change the results, and it was not included in the final models. Data analysis was performed using the Stata version 9 statistical software (StataCorp, College Station, TX, USA).

## Results

By design, the mean age (years) of the study population was similar in both groups: 53.41 ± 12.78 in cases and 53.83 ± 12.54 in controls (data not shown). The known BC factors associated with a reduction (higher parity and lactation) and with an increased risk (early menarche, older age at first childbirth, and higher body mass index after menopause) were confirmed in this population (Table 1). Family history of BC was absent among controls and is not included in Table 1.

We found significant negative correlations between age at menarche and the urinary concentrations of MBP and MiBP among controls. In contrast, current age and parity showed significant positive correlations with the urinary concentrations of MEHHP and MECPP, respectively, as well as parity with MEOHP. We detected no other significant correlations between phthalate metabolite concentrations and BC risk factors among controls (Table 2).

All samples had detectable concentrations of MEP, MBP, MEHHP, MEOHP, and MECPP. Concentrations were most likely to be undetectable for MEHP (82% of samples). MEP geometric mean concentrations were significantly higher in cases than in controls. Conversely, controls had significantly higher geometric mean MBP, MCPP, and MEOHP levels than did cases. Similar patterns were observed after stratifying by menopausal status (Table 3). Significant (*p* < 0.05) correlation coefficients (> 0.6) were observed among the DEHP metabolites as well as between MCPP [a minor metabolite of DBP and of other phthalates (Calafat et al. 2006)] and MBP (the major metabolite of DBP); other metabolites showed lower correlations [see Supplemental Material (doi:10.1289/ehp.0901091)].

MEP urinary concentrations were significantly associated with BC, with a significant test for lineal trend. In contrast, significant negative associations were observed with MiBP, MBzP, and MCPP. Neither the sum of DEHP metabolites nor the specific DEHP metabolite concentrations showed an association with BC, except for MECPP, which showed borderline significance after adjusting for reproductive covariates and other phthalates (Table 4).

When stratified by menopausal status, the increased BC risk observed for the highest versus lowest tertile of MEP was significantly higher only in premenopausal women, and the association remained when adjusted for other phthalate metabolites [odds ratio (OR) = 4.13; 95% confidence interval (CI), 1.60–10.70; *p* for trend < 0.004]; this was also the case for the negative associations with BC observed for the highest versus lowest tertile of MBzP (OR = 0.22; 95% CI, 0.08–0.61; *p* for trend < 0.006) and for the highest versus lowest tertile of MCPP (OR = 0.18; 95% CI, 0.05–0.59; *p* for trend < 0.003) (Table 5).

## Discussion

This study suggests for the first time that urinary concentrations of some phthalate metabolites may be related to BC independent of other known risk factors: MEP, the main DEP metabolite, is significantly associated with incident BC in premenopausal women, and the metabolites of BBzP (MBzP) and of DOP and other phthalates (MCPP) are inversely associated with BC.

Two recent epidemiologic reports involving men attending an infertility clinic showed that sperm DNA damage was associated with urinary concentrations of MEP but not of MBP or MBzP (Duty et al. 2003; Hauser et al. 2007). Other phthalates and/or their metabolites have been reported to damage DNA, as assessed by the alkaline comet assay (single-cell gel electrophoresis). DNA damage was detected in human lymphocytes exposed to DEHP and MEHP (Anderson et al. 1999). Also, DBP and DiBP were shown to be genotoxic in human epithelial cells of the upper aerodigestive tract, mucosal cells, and lymphocytes (Kleinsasser et al. 2000). These studies suggest that MEP and other phthalates have the potential to induce DNA damage and increase cancer risk; however, further research is needed to fully characterize the genotoxic effects of phthalates on human breast cells.

The epigenetic effects of phthalates, such as DNA methylation, might explain the negative associations of MBP and MBzP with BC observed in this study. BBzP and DBP, parent compounds of MBzP and MBP, respectively (MBP is also a minor metabolite of BBzP), led to demethylation of estrogen receptor α promoter–associated CpG islands, producing a growth inhibitory effect on human MCF-7 BC cells (Kang and Lee 2005), thus reducing BC risk.

Another potential underlying mechanism to explain the mentioned negative associations is related to the effects of phthalates on peroxisome proliferator–activated receptors (PPARs) and the key role these ligand-activated transcription factors have in the proliferation and differentiation of BC cell lines and in breast development. PPARγ is associated with differentiation, increased lipid accumulation, and inhibition of BC cell proliferation (Elstner et al. 1998; Mueller et al. 1998), and available evidence shows that DBP/MBP and BBzP/MBzP have moderate but consistent associations with PPARγ activation (Bility et al. 2004; Feige et al. 2007; Hurst and Waxman 2003). In this context, further information is needed to explain the negative association found in this study regarding MCPP, a nonspecific metabolite of several phthalates, including DOP and DBP (Calafat et al. 2006), and BC.

Regarding DEHP metabolites, we found no association with BC in this study. These findings may result from the opposing effects that individual phthalates and/or metabolites could have. For example, MEHP activated both human PPARα and PPARγ but not PPARβ; the former PPARs have opposing functions in the breast epithelial cell line MCF-7, so the net effect of DEHP exposure would result from a complex series of competitive underlying mechanisms. In this context, MBP acted as an antagonist for both PPARγ and PPARβ (Venkata et al. 2006). Studies summarizing the effects of each parent phthalate and each metabolite, whether they are estrogenic or androgenic, and whether they activate or inhibit the various PPARs have been published (Corton and Lapinskas 2005; Hatch et al. 2008).

Deodorants, fragrances, creams, and nail polishes, among other personal care products, may contain phthalates, including DEP, the parent compound of MEP, as well as DBP and BBzP, the parent compounds of MBP and MBzP, respectively (Chingin et al. 2009; Cosmetic Ingredient Review 2003; Health Care without Harm 2002; Houlihan et al. 2002; Koo and Lee 2004; Scientific Committee on Cosmetic Products and Non-food Products 2002). DEP has been found in a high proportion (28–71%) of the personal care products, 57–72% of perfumes, and 25% of deodorants tested in some studies. DBP was detected in 24–75% of personal care products, 26% of perfumes, and 67–90% of the nail polishes, and DOP (parent compound of MCPP) was detected in 21% of deodorants. In contrast, BBzP and DEHP were not detected in most deodorants and hair products and in less than one-third of all products (Chingin et al. 2009; Health Care without Harm 2002; Houlihan et al. 2002; Koo and Lee 2004). DEP, DBP, DiBP, DEHP, and BBzP have also been detected in foods (Wormuth et al. 2006), and DBP has been found in water from plastic bottles (Cao 2008). Some of these phthalates are used in the production of enteric coatings for drugs (Hauser and Calafat 2005).

The main route of exposure to DEP is dermal (Agency for Toxic Substances and Disease Registry 1995; Api 2001; Mint et al. 1994; Scott et al. 1987). The application of a cream containing DEP and DBP on the entire body enabled detection of their main metabolites in urine, indicating a fast and regular absorption through the skin (Janjua et al. 2007, 2008). In addition, a steep relationship between urinary MEP levels and the reported use of personal care products in the 48 hr before urine collection was observed in a group of men in the United States (Duty et al. 2005), and increasing urinary concentrations with the use of DEP-containing products, without statistical dose–response relationship, have been recently observed in a small group of Israeli women (Berman et al. 2009).

Differences in the phthalate metabolite profiles among women from various countries have been observed (Berman et al. 2009; Hogberg et al. 2008; Huang et al. 2007). For the present study, the urinary concentrations of MBP, MiBP, MEHP, MEHHP, and MEOHP found in the cases and controls were higher than those reported for females in the general U.S. population from the 2001–2002 National Health and Nutrition Examination Survey (MBP = 21.7, MiBP = 2.87, MEHP = 4.53, MEHHP = 19.7 μg/g creatinine) and other specific groups of U.S. women (Adibi et al. 2008; CDC 2005; Hines et al. 2009; Peck et al. 2010; Swan et al. 2005; Wolff et al. 2008). Currently, limited information is available regarding the presence of phthalates in personal care and other products (e.g., drugs) to estimate their relative contribution to the total body burden of phthalates.

As for the methodological strengths of our project, > 90% of BC incident cases during the study period in the study area were included, as well as a representative sample of healthy women drawn from the population in which the cases occurred, which adds external validity to the study. This is supported by the confirmation of the presence of known BC factors (e.g., lactation, parity) in this population (World Cancer Research Fund/American Institute for Cancer Research 2007). Also, the very high rates of participation reduced the possibility that different characteristics (potential or observed) among the very few nonparticipants versus participants might have influenced results. The possibility of a differential measurement error is very low because phthalate exposure was evaluated in biological samples and so did not depend on women’s reports, and the laboratory assessment was blind in relation to case/control status. Moreover, urine samples for phthalate determination were obtained in all cases before treatment began, so it is unlikely that phthalate exposure in cases was influenced by cancer treatment. However, further information is needed regarding disease status that might be reflected in changes of phthalate metabolite concentrations.

Our study also has several potential limitations. Although urinary concentrations of phthalate metabolites are the most common strategy for assessing human exposure to phthalates and are an integral measurement of exposure through multiple sources and routes, an important limitation of this study relates to the fact that a single measurement does not allow evaluation of cumulative exposure or exposure windows. Therefore, the assumption underlying our interpretation is that the urinary phthalate concentrations observed in these women predict their steady-state concentrations, if we consider that the use of personal care products may be fairly constant over time. However, further studies are needed to characterize the magnitude and profile of exposure in order to obtain a more valid predictive biomarker of phthalate exposure. Also, because of the small sample size involving stratification of pre- and postmenopausal women, additional studies are needed to assess potential differences of the associations between phthalate exposure and BC by menopausal status.

## Conclusions

Our results show for the first time that exposure to DEP, as assessed by urinary MEP concentrations, may be associated with an increase in BC risk, whereas the exposure to other phthalates, measured by the urinary concentrations of MBzP (BBzP) and MCPP (DOP and other phthalates), were negatively associated with BC. The findings require confirmation to exclude the possibility that these parent/metabolite phthalates are surrogates of unrecognized lifestyle or dietary BC risk factors.

The various sources and levels of exposure to relevant phthalates present in cosmetics and other personal care products deserve further assessment, particularly at critical windows of exposure, such as adolescence. Also, the biological mechanisms warrant clarification.

## Figures and Tables

**Table 1:** Age-adjusted ORs for known BC risk factors.

Factor	Cases/controls (*n* = 233/221)	OR (95% CI)
Age of menarche (years)		
> 12	129/145	1.00
≤ 12	103/76	1.52 (1.03–2.23)
Parity (no.)		
Nulliparous	20/8	1.00
1–3	91/73	0.52 (0.22–1.26)
4–6	74/71	0.41 (0.17–0.99)
> 6	47/69	0.23 (0.09–0.60)*
Age at first birth (years)		
< 19	52/79	1.00
19–21	62/64	1.47 (0.90–2.41)
≥ 22	99/70	2.15 (1.35–3.42)
Nulliparous	20/8	3.77 (1.55–9.21)*
Lactation		
No	70/26	1.00
< 1 year	91/78	0.44 (0.25–0.76)
≥ 1 year	68/117	0.21 (0.12–0.36)*
Body mass index		
Premenopause		
< 25	28/24	1.00
25–29.99	35/23	1.33 (0.62–2.84)
≥ 30	25/27	0.83 (0.38–1.81)
Postmenopause		
< 25	14/36	1.00
25–29.99	57/47	3.25 (1.56–6.78)
≥ 30	74/64	3.14 (1.55–6.40)**

Some numbers do not equal the total sample size because of missing values.

* *p*-Value for trend < 0.05.

** *p*-Value for trend = 0.056.

**Table 2:** Correlation coefficients between BC covariates and urinary phthalate metabolites (μg/g creatinine) among controls (*n* = 221).

	Phthalates (parent/metabolite)								
Breast cancer covariate	DEP/MEP	DBP/MBP	DiBP/MiBP	BBzP/MBzP	DOP^a^/MCPP	DEHP/MEHP	DEHP/MEHHP	DEHP/MEOHP	DEHP/MECPP
Age (years)	−0.03	−0.02	−0.12	−0.07	0.06	0.07	0.18*	0.11	0.16*
Age at menarche (years)	0.05	−0.22*	−0.13*	−0.05	−0.13	−0.10	−0.01	−0.01	−0.03
Parity (number)	−0.05	0.01	0.02	−0.01	0.07	0.02	0.20*	0.17*	0.18*
Age at first birth (years)	−0.12	−0.08	−0.07	−0.01	0.01	0.11	0.06	0.04	0.04
Body mass index (kg/m^2^)	−0.01	0.02	0.02	−0.05	−0.08	−0.07	0.05	−0.02	0.03

a Includes other phthalates.

* *p* < 0.05.

**Table 3:** Urinary phthalate metabolites (μg/g creatinine) in the study population by menopausal status [geometric mean (95% CI)].

	All			Premenopause			Postmenopause		
Parent compound/metabolite	Cases (*n* = 233)	Controls (*n* = 221)	*p*-Value	Cases (*n* = 88)	Controls (*n* = 74)	*p*-Value	Cases (*n* = 145)	Controls (*n* = 174)	*p*-Value
DEP/MEP	169.58 (141.92–202.64)	106.78 (91.25–124.96)	< 0.001	184.77 (138.93–245.73)	111.69 (84.53–147.57)	0.007	160.98 (127.88–202.63)	104.40 (86.11–126.56)	0.002

DBP/MBP	62.98 (56.06–70.76)	82.47 (72.67–93.60)	0.001	57.56 (47.63–69.55)	81.61 (65.61–101.51)	0.008	66.52 (57.33–77.18)	82.91 (70.85–97.03)	0.023

DiBP/MiBP	7.81 (6.93–8.81)	8.85 (7.95–9.84)	0.065	8.31 (6.85–10.09)	9.99 (8.42–11.85)	0.084	7.53 (6.45–8.78)	8.32 (7.27–9.52)	0.167

BBzP/MBzP	5.43 (4.81–6.13)	6.27 (5.38–7.31)	0.072	5.29 (4.42–6.34)	7.22 (5.67–9.20)	0.020	5.51 (4.68–6.49)	5.84 (4.80–7.10)	0.327

DOP and other phthalates/MCPP	2.68 (2.43–2.95)	4.07 (3.66–4.54)	< 0.001	2.57 (2.23–2.95)	3.92 (3.22–4.78)	< 0.001	2.75 (2.41–3.14)	4.15 (3.64–4.73)	< 0.001

DEHP/MEHP	5.13 (4.45–5.90)	5.09 (4.47–5.78)	0.467	6.13 (4.95–7.60)	5.42 (4.25–6.91)	0.224	4.60 (3.82–5.53)	4.93 (4.23–5.73)	0.285

DEHP/MEHHP	49.10 (43.65–55.23)	48.71 (44.10–53.81)	0.460	45.00 (37.05–54.66)	41.22 (33.81–50.24)	0.266	51.76 (44.60–60.07)	52.99 (47.44–59.19)	0.401

DEHP/MEOHP	28.11 (25.01–31.59)	33.37 (30.29–36.77)	0.014	27.47 (22.8–33.10)	30.15 (24.98–36.40)	0.244	28.50 (24.50–33.15)	35.12 (31.41–39.26)	0.014

DEHP/MECPP	87.10 (78.37–96.80)	79.16 (72.06–86.97)	0.093	82.74 (69.35–98.72)	70.09 (58.39–84.14)	0.099	89.85 (78.66–102.63)	84.17 (75.58–93.73)	0.225

Sum of metabolites	174.35 (156.58–194.15)	169.69 (154.60–186.26)	0.355	165.65 (138.65–197.91)	149.82 (124.7–180.01)	0.219	179.86 (156.94–206.12)	180.67 (162.60–200.76)	0.479

Percentage of samples below LOD: among cases, 0% for MEP, MBP, MEHHP, MEOHP, and MECPP, 3% for MiBP, 2% for MBzP, 3% for MCPP, and 18% for MEHP; among controls, 0% for MEP, MBP, MEHHP, MEOHP, and MECPP, 3% for MiBP, 10% for MBzP, 2% for MCPP, and 17% for MEHP.

**Table 4:** Adjusted ORs for urinary phthalate metabolites and BC.

			OR (95% CI)	
Phthalate tertile (μg/g creatinine)	Median in controls	Cases/controls	Adjusted for BC covariates^a^	Adjusted for BC covariates and phthalate metabolites^b^
DEP/MEP				
9.40–56.18	32.79	56/74	1.00	1.00
56.19–181.35	99.76	72/74	1.26 (0.77–2.05)	1.42 (0.85–2.38)
181.36–18985.50	386.75	103/73	1.94 (1.21–3.12)	2.20 (1.33–3.63)
*p*-Value for trend			0.005	0.003

DBP/MBP				
6.21–52.55	35.84	97/74	1.00	1.00
52.55–113.69	72.62	87/74	0.89 (0.57–1.38)	1.08 (0.66–1.78)
113.70–1746.03	179.30	47/73	0.46 (0.28–0.76)	0.85 (0.47–1.57)
*p*-Value for trend			0.001	0.511

DiBP/MiBP				
0.23–7.44	4.90	105/74	1.00	1.00
7.45–12.07	9.55	60/74	0.61 (0.38–0.97)	0.59 (0.35–0.98)
12.08–86.22	17.40	66/73	0.68 (0.43–1.07)	0.73 (0.43–1.24)
*p*-Value for trend			0.125	0.365

BBzP/MBzP				
0–5.18	2.85	113/74	1.00	1.00
5.19–10.79	7.23	68/74	0.59 (0.38–0.93)	0.60 (0.37–0.98)
10.80–258.62	16.45	50/73	0.46 (0.29–0.74)	0.46 (0.27–0.79)
*p*-Value for trend			0.002	0.008

DOP and other phthalates/MCPP				
0.22–2.83	2.04	119/74	1.00	1.00
2.84–5.28	3.73	72/74	0.69 (0.44–1.07)	0.76 (0.47–1.23)
5.29 –193.91	8.25	40/73	0.35 (0.21–0.57)	0.44 (0.24–0.80)
*p*-Value for trend			< 0.001	0.007

DEHP/MEHP				
0.22–3.42	2.06	81/74	1.00	1.00
3.43–7.51	5.33	67/74	0.85 (0.53–1.36)	1.03 (0.62–1.69)
7.52–257.08	12.28	83/73	0.99 (0.63–1.57)	1.23 (0.75–2.01)
*p*-Value for trend			0.904	0.383

DEHP/MEHHP				
2.69–35.61	24.93	90/74	1.00	1.00
35.62–63.38	47.47	53/74	0.61 (0.38–0.99)	0.77 (0.46–1.28)
63.39–1014.60	95.58	88/73	1.01 (0.64–1.58)	1.37 (0.84–2.24)
*p*-Value for trend			0.650	0.106

DEHP/MEOHP				
2.10–23.90	17.39	108/74	1.00	1.00
24.91–43.10	32.95	51/74	0.49 (0.31–0.79)	0.60 (0.36–1.00)
43.11–1230.94	64.62	72/73	0.66 (0.42–1.04)	0.84 (0.52–1.36)
*p*-Value for trend			0.130	0.651

DEHP/MECPP				
11.59–57.88	42.02	69/74	1.00	1.00
57.89–97.67	74.56	73/74	1.04 (0.65–1.67)	1.27 (0.77–2.10)
97.68–1742.92	155.88	89/73	1.31 (0.82–2.08)	1.68 (1.01–2.78)
*p*-Value for trend			0.222	0.047

Sum of metabolites				
18.56–120.79	88.09	82/74	1.00	1.00
120.80–207.46	161.62	62/74	0.79 (0.49–1.27)	0.94 (0.57–1.56)
207.47–4,193.90	317.41	87/73	1.09 (0.69–1.71)	1.41 (0.86–2.31)
*p*-Value for trend			0.539	0.114

a Adjusted for current age, age of menarche, parity, and menopausal status.

b Adjusted for current age, age of menarche, parity, and menopausal status plus phthalate metabolites: DEHP metabolites were adjusted for non-DEHP metabolites; MEP, MBP, MiBP, BBzP, and MCPP were adjusted for themselves plus the sum of DEHP metabolites.

**Table 5:** Adjusted ORs for MEP, MBzP, and MCPP urinary concentrations and BC by menopausal status.

	Premenopause		Postmenopause	
Phthalate tertile (μg/g creatinine)	Cases/controls	OR (95% CI)^a^	Cases/controls	OR (95% CI)^a^
DEP/MEP				
9.40–56.18	16/24	1.00	40/50	1.00
56.19–181.35	31/29	1.84 (0.73–4.6)	41/45	1.32 (0.69–2.53)
181.36–18985.50	40/21	4.13 (1.60–10.7)	63/52	1.84 (0.99–3.42)
*p*-Value for trend		0.004		0.060

BBzP/MBzP				
0–5.18	44/21	1.00	69/53	1.00
5.19–10.79	26/27	0.36 (0.15–0.89)	42/47	0.71 (0.38–1.30)
10.80–258.62	17/26	0.22 (0.08–0.61)	33/47	0.61 (0.31–1.19)
*p*-Value for trend		0.006		0.169

DOP and other phthalates/MCPP				
0.22–2.83	48/26	1.00	71/48	1.00
2.84–5.28	30/22	0.83 (0.34–2.03)	42/52	0.65 (0.35–1.18)
5.29–193.91	9/26	0.18 (0.05–0.59)	31/47	0.52 (0.25–1.08)
*p*-Value for trend		0.003		0.106

a Adjusted for current age, age of menarche, parity, and phthalate metabolites: DEHP metabolites were adjusted for non-DEHP metabolites; MEP, MBP, MiBP, BBzP, and MCPP were adjusted for themselves plus the sum of DEHP metabolites.
